# Depthwise microbiome and isotopic profiling of a moderately saline microbial mat in a solar saltern

**DOI:** 10.1038/s41598-020-77622-w

**Published:** 2020-11-26

**Authors:** Varun Paul, Yogaraj Banerjee, Prosenjit Ghosh, Susheel Bhanu Busi

**Affiliations:** 1 Department of Geosciences, Mississippi State University, Starkville, MS 39762 USA; 2 Interdisciplinary Centre for Water Research, Indian Institute of Science, Bangalore, 560012 India; 3 Centre for Earth Sciences, Indian Institute of Science, Bangalore, 560012 India; 4 Luxembourg Centre for Systems Biomedicine, University of Luxembourg, 4362 Esch-sur-Alzette, Luxembourg

**Keywords:** Microbial ecology, Stable isotope analysis, Biogeochemistry

## Abstract

The solar salterns in Tuticorin, India, are man-made, saline to hypersaline systems hosting some uniquely adapted populations of microorganisms and eukaryotic algae that have not been fully characterized. Two visually different microbial mats (termed ‘white’ and ‘green’) developing on the reservoir ponds (53 PSU) were isolated from the salterns. Firstly, archaeal and bacterial diversity in different vertical layers of the mats were analyzed. Culture-independent 16S rRNA gene analysis revealed that both bacteria and archaea were rich in their diversity. The top layers had a higher representation of halophilic archaea *Halobacteriaceae*, phylum *Chloroflexi*, and classes *Anaerolineae*, *Delta*- and *Gamma*- *Proteobacteria* than the deeper sections, indicating that a salinity gradient exists within the mats. Limited presence of *Cyanobacteria* and detection of algae-associated bacteria, such as *Phycisphaerae*, *Phaeodactylibacter* and *Oceanicaulis* likely implied that eukaryotic algae and other phototrophs could be the primary producers within the mat ecosystem. Secondly, predictive metabolic pathway analysis using the 16S rRNA gene data revealed that in addition to the regulatory microbial functions, methane and nitrogen metabolisms were prevalent. Finally, stable carbon and nitrogen isotopic compositions determined from both mat samples showed that the δ^13^C_org_ and δ^15^N_org_ values increased slightly with depth, ranging from − 16.42 to − 14.73‰, and 11.17 to 13.55‰, respectively. The isotopic signature along the microbial mat profile followed a pattern that is distinctive to the community composition and net metabolic activities, and comparable to saline mats in other salterns. The results and discussions presented here by merging culture-independent studies, predictive metabolic analyses and isotopic characterization, provide a collective strategy to understand the compositional and functional characteristics of microbial mats in saline environments.

## Introduction

Solar salterns are natural or man-made, extreme hypersaline environments associated with the production of salt. The coastal solar saltern in Tuticorin, India, is the second largest salt mining industry in the country. These salterns typically consist of three successive systems in a grid pattern, separated by mud ridges: (i) reservoir, where subsurface brine or seawater is stored, (ii) condenser, where the stored saline water is concentrated through evaporation, and (iii) crystallizer, where precipitation of salts and harvesting takes place. Solar salterns throughout the world have been studied for extreme, salt-tolerant organisms that they support, including microorganisms, brine shrimp (*Artemia salina*) and beetles^1–3^. The microbial life in these environments have unique adaptations to the extreme osmotic conditions, which have been commercially and scientifically exploited in areas such as pharmaceutical, food production, environmental remediation and astrobiology^4–7^.

The diversity and richness of the microorganisms found in these environments have been an area of steady interest, and novel species are routinely identified through culture-dependent and -independent studies^8–12^. These salterns sometimes host organized, layered, millimeter-to-centimeter-thick consortium of various microorganisms in the form of microbial mats^13,14^. The microbial mats are usually dominated by photosynthetic algae or *Cyanobacteria*, with a diverse community of microorganisms and associated metabolisms^15,16^. Nutrients are rapidly shuttled between the different members and the ‘microgradients’ they produce, resulting in a dynamic, yet self-sustaining ecosystem. While the microbial population is generally well-studied, there is still a lot more knowledge to be gained about the functional capacity within the gradient mats^14^. However, the flourishing high-throughput sequencing capabilities, and the ‘omics’-based approach to understand both phylogeny and metabolic pathways, have created a more precise and detailed understanding of the functioning of these ecosystems^7,17–19^. Albeit limited in comparison to whole-genome shotgun metagenomics, predictive functional traits using 16S rRNA gene data is a useful tool that provides a representation of the dominant metabolic activities relative to the community members^20^; however, their application in halophilic studies have so far been limited^21^. Since 16S rRNA genes are primarily used for taxonomic assignments, the use of this data to predict metabolic pathways should be treated with caution as the functional genes themselves are not identified. In addition to bulk mat sequencing, several reports have also highlighted the importance of a depth-wise, stratigraphic analysis of the microbial mats to understand the cycling of key elements^22–25^.

Studies have also focused on profiling the isotopic characteristics of these mats to aid in deciphering the functional capabilities of the microorganisms^25–28^. Stable isotope profiles can give clues as to the dominant microbial metabolism and mineralogical influences at different layers within the mat^29^. This information, in turn, has important consequences in understanding past environments and climatic conditions^30–32^.

Very few studies targeting microbial diversity and metabolic functions in saline and hypersaline mats from the Indian subcontinent have been undertaken to date^12,33^. While some efforts have targeted specific microbial members, such as archaea, *Cyanobacteria*, and *Actinomycetes*^34–37^, a complete, depth-wise amplicon and metagenomic analysis is lacking. Additionally, the application of stable isotope systematics to understand the growth of microbial mat and microbialite formation from this region, especially in hypersaline environments, are very limited^38–41^. Almost all of these studies are related to Cambrian time period or older. On the other hand, microbial mats of salterns from other parts of the world, such as in Israel, Slovenia and Guerrero Negro (GN) in Baja California, have been extensively studied for their biogeochemical characteristics^22,42–45^.

The goals of the present study are three-fold: (i) perform a depth-wise 16S ribosomal RNA amplicon sequencing of the microbial mats isolated from the reservoir pond of the saltern to understand the spatial distribution of their bacterial and archaeal population, (ii) apply predictive metabolics using 16S rRNA gene data to differentiate between dominant metabolic pathways, and (iii) determine the relationship between the microbial mat population, predicted functions, and the carbon and nitrogen isotopic signatures.

## Methodology

### Site description and sample collection

**Figure 1:**
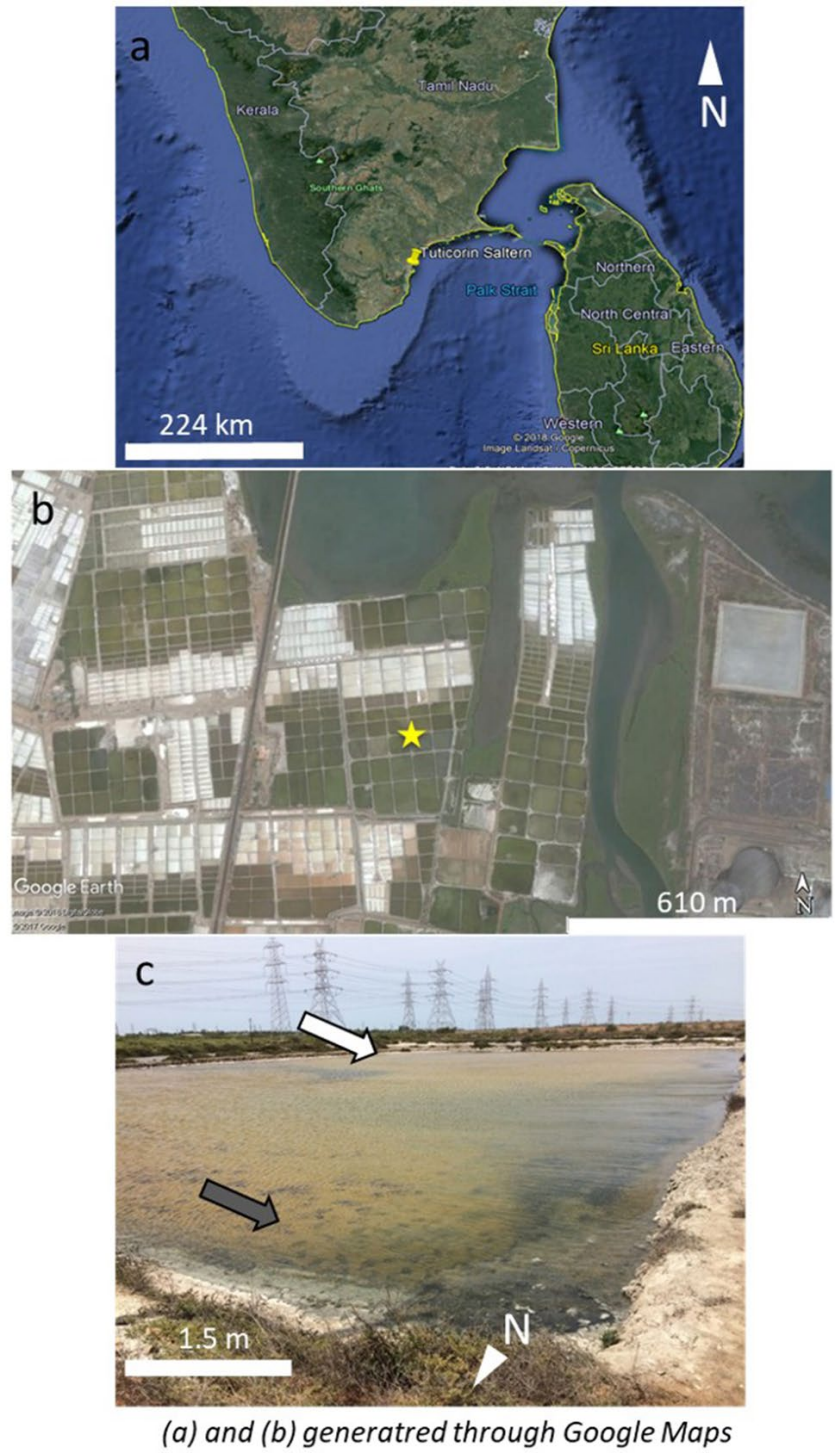
Google Map showing location of Tuticorin (yellow pin), Tamil Nadu, South India (**a**), Google map location of the reservoir ponds (yellow star) from which the microbial mat samples was collected (**b**), and the reservoir pond (**c**) showing the white (white arrow) microbial mat in the far end and green (grey arrow) microbial mat coating the bottom surface of the pond. (**a**) and (**b**) obtained from Google maps (https://www.google.com/maps/) and scale bars added in Microsoft PowerPoint.

**Figure 2:**
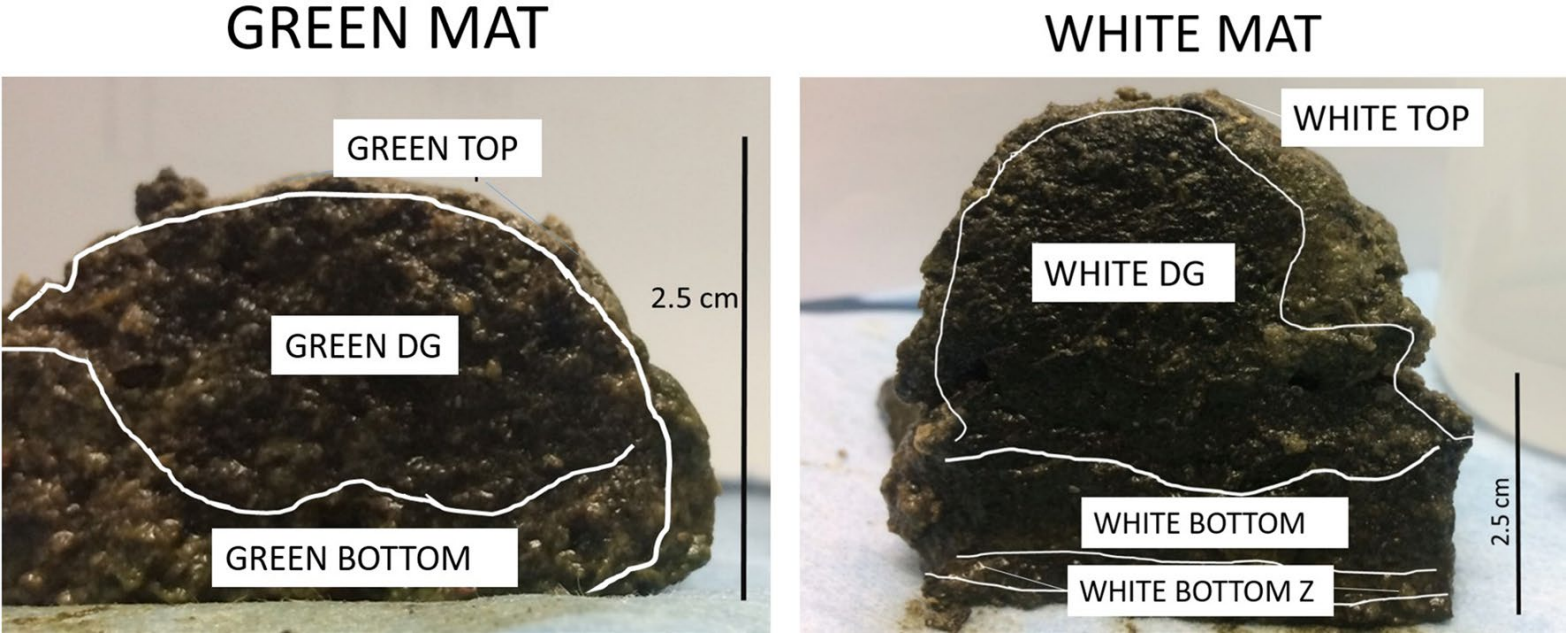
Cross-section of the green and white mats isolated from the reservoir ponds. The different layers sectioned based on visual observations and their assigned designations in both mats are shown.

Microbial mat samples were collected from a reservoir pond in a salt harvesting facility in Tuticorin, Tamil Nadu, India (Fig. 1) in May 2017 from 10:00 AM to 01:00 PM (IST). The air temperature was 33.8 °C at the time of sample collection. The water in the reservoir pond had a high salinity, 53 practical salinity units (PSU). The facility also consisted of condenser and crystallizer ponds, where the salt is sequentially concentrated. The highest salinity up to 140 PSU was recorded in the crystallizer ponds. The dense microbial mat found in the reservoir ponds were not observed in the condenser and crystallizer ponds, likely because the extreme saline conditions inhibited the formation of the mats in the latter settings. Among the microbial mats found in the reservoir ponds, two distinct colorations were observed on the top surface layers of the mat: green and white. The reservoir ponds are constructed along a gradient to allow water flow naturally. The white mat was collected from a higher elevation along this gradient, making the location more prone to water fluctuations than the green mat, which was sampled from a lower gradient. Thus, the former mat type might have experienced periods of dryness when the water level was low. Both mats were completely submerged under water during collection, but it is suspected that the coloration on top of the white mat was likely due to the partial exposure above the water. This would have led to drying and possible bleaching, resulting in the white color^46^. The dried-up material causing white coloration washed off during transportation and hence are not visible in Fig. 2. Whole sections of both mat types were carefully removed, saved in a solution of RNAlater (Thermo Fisher Scientific, Waltham, MA, USA), and kept frozen during transportation. The samples were then thawed in a clean laboratory, and partitioned using a sterile laboratory spatula.

The mats had the consistency of a thick sponge, but were easily dissected without application of much force. Both the green and white mats were sectioned into different near-horizontal partitions based on visual and textural changes (Fig. 2). Broadly, both mats were almost identical except for the green or white layer on the top. In the green mat, three sections were identified and isolated: (i) the top green crust-like layer (Green-TOP) that stretched across the entire profile of the mat, extending about 0.1–0.2 cm from the top surface, (ii) a middle darker green, fibrous material (Green-DG) that was the thickest (0.4–2.0 cm) amongst all layers, and (iii) a bottom horizontal layer (Green-BOT) that was made of at least two or three sheets of smaller layers, each about 0.3 cm thick. The total thickness of this bottom layer ranged from 0.5 to 1.5 cm. As mentioned earlier, the white mat was similar to the green mat, but all layers were generally thicker. White-TOP and White-DG were the top and dark green sections of the white mat, respectively. The bottom layers, which had a more pronounced sheet-like texture, was easily sectioned horizontally, and was further divided into two zones based: White-BOT, which had porous features, and White-BTZ, which had highly compacted organic material. A small section of the white material was reacted with 5% HCl and the lack of effervescence indicated absence of carbonate minerals. A simple salinity (electrical conductivity) measurement was conducted for the different vertically sectioned mat samples. 10 g of each section of the dried (50 °C overnight) mat sample was added to 10 ml of de-ionized water, mixed and allowed to settle for 30 min^47^. The conductivity values were then measured using a Hanna Conductivity Probe (HI9033). Water samples were collected from the reservoir ponds from which the mats were obtained, the condenser, and crystallizer ponds for δ^18^O analysis.

### Culture-independent 16S rRNA gene sequencing

#### DNA extraction

The sectioned samples were stored in RNAlater, frozen and shipped to the University of Missouri DNA Core Facility, where DNA extraction, library preparation and sequencing were performed. High-quality DNA was extracted using Qiagen PowerSoil DNA extraction kit according to the manufacturer’s instructions and eluted in 100 µL of buffer (Qiagen, Carlsbad, CA). DNA yields were quantified via fluorometry (Qubit 2.0, Life Technologies, Carlsbad, CA) using quant-iT BR dsDNA reagent kits (Invitrogen, Carlsbad, CA) and DNA was diluted to a final concentration of 3.81 ng/μl with sterile water in 96-well plates to a uniform concentration and volume for library preparation.

#### 16S rRNA gene library preparation and sequencing

Bacterial and archaeal 16S rRNA gene amplicons were generated via amplification of the V4 hypervariable region of the 16S rRNA gene using the universal primers (U515F/806R), flanked by standard Illumina adapter sequences. A single forward primer and indexed reverse primers were used in all reactions. The primer sequences were as follows, *forward*: GTGCCAGCMGCCGCGGTAA and *reverse*: GGACTACHVGGGTWTCTAAT^48^. PCR was performed in 50 µL reactions containing 100 ng metagenomic DNA, primers (0.2 µM each), dNTPs (200 µM each), and Phusion high-fidelity DNA polymerase (1U). The PCR reaction were as follows: 3:00 min at 98 °C, 25 cycles of (0:15 min at 98 °C + 0:30 min at 50 °C + 0:30 min at 72 °C), and a final extending step for 7:00 min at 72 °C. For 16S rRNA gene amplicon sequencing, the Illumina MiSeq platform in conjunction with a set of 96 bar-coded primers was used. Amplicons were pooled by mixing 5 µL/reaction, and purified by adding 50 µL of this mixture to 50 µL of Axygen Axyprep Mag PCR clean-up beads. Following a 15-min incubation at room temperature, the products were repeatedly washed with 80% ethanol. These washes resulted in a dry pellet that was allowed to resuspend in 32.5 µL elution buffer followed by a two-minute room temperature incubation, before finally being placed for five minutes on a magnetic stand. An Advanced Analytical Fragment Analyzer automated electrophoresis system was used to analyze the final amplicon pool. Quantification of the amplicon pool was achieved using quant-iT HS dsDNA reagent kits. Before sequencing on the MiSeq instrument, the samples were diluted according to Illumina’s standard protocol.

#### Informatics analysis

The initial bioinformatics steps were performed at the University of Missouri Informatics Research Core Facility as part of a standard protocol. Demultiplexing the samples was achieved by using Qiime v1.9 command split_libraries_fastq.py^49^. Primer sequences at both ends of the contig were deleted using Cutadapt^50^. The expected number of errors flag was used and set at 0.5 as identified and recommended by the Usearch manual, whereupon the usearchfastq_filter command (http://drive5.com/usearch/manual/cmd_fastq_filter.html) was used for trimming^51^. Briefly, sequences that exceeded 292 base length were deleted following trimming for base quality of 31. Additionally, we determined the distribution of read lengths, and found 7 reads to be below 248 bp in length, whereas the remaining reads had an average length of 251 bp. Based on this, we trimmed reads below a length of 248 bp. FLASH was then used to merge the paired-end read with a minimum overlap of 30 bp and a maximum overlap of 292 bp^52^. The outputs from all samples were subjected to clustering by combining the generated and processed data into a single file. The uparse method (http://www.drive5.com/uparse/) clustered contigs with 97% identity and remove chimeras^53^. The SILVA database v128 of 16S rRNA gene sequences was used to allocate taxonomy to selected OTUs based on a minimum identity of 75% as suggested by the PyNast alignment script implemented in Qiime v1.9^54^. We have provided the full OTU fasta file and taxonomic assignment information in this link for perusal: https://github.com/vrnpaul/Saltern-Krona-data. Alpha diversity index parameters including Chao1, Shannon, and observed species were computed using QIIME. Krona plots using an Excel template were applied as a tool to visualize and interact with the results of the taxonomic composition generated^55^. Alpha-diversity boxplots were generated using the *phyloseq*R package with the input of OTU biom table, taxonomic assignments and the tree information. Samples with a read count below 5,000 were removed from the analysis due to insufficient rarefaction, due to which the ‘Green-DG’ sample was removed from the analyses. The average read counts for all samples was 47,882. Principal Coordinate Analyses (PCoA) were performed in PAST (PAleontological STatisitics, version 3.2)^56^. Simultaneously, a scree plot was generated using the Rscript chemometrics under the *metaboanalyst* package to identify the principal coordinates for the figures. Heatmap was generated using the *plotHeatMap* function from the same package along with the *hclust* function from the *stat* package. For the predictive metabolic potential, the Tax4Fun R package was used to identify congruent microbial pathways based on KEGG (Kyoto Encyclopedia of Genes and Genomes) orthology^57,58^. While the program has been applied before to predict metabolisms based on OTU in for environmental samples^59^, the applicability has been shown to be more suited for human microbiome functional predictions^60^. For the heatmaps, Euclidean distance was used as the similarity measure, while Ward’s clustering algorithm accounting for average linkage was used to create the Dendrogram. All OTUs and KEGG pathways were normalized using the *cumnorm* function (cumulative sum-scaling normalization) to account for the zero-inflated large dataset, including the exclusion of features with a relative abundance below 0.00001%.

### Isotopic analysis

For the stable isotope investigations, the mat samples were sectioned along the same layers as that used for microbial sequencing. Possible inorganic remains were leached out using 10% HCl, and the remaining bulk organic matter was rinsed with de-ionized water and kept in an oven (70 °C) overnight for drying. The carbon and nitrogen isotope ratios of the bulk organic matter from individual microbial mat layers were then measured following the protocol described in Kaushal et al.^61^. Briefly, for the carbon isotope analyses, 170–180 μg of the dried sample powder was weighed and packed in tin capsules. The powder-filled capsules were then analysed using elemental analyser FLASH 2000 (Thermo Scientific) attached with an isotope ratio mass spectrometer (IRMS) Delta V Advantage (Thermo Scientific, Bremen) at the Oasis laboratory, Indian Institute of Science, Bangalore. Each sample was analysed in duplicates for assigning average value and understanding heterogeneity of the isotopic composition. The results are expressed as delta (δ) values in per mil (‰), with respect to the Vienna Pee Dee Belemnite (VPDB) standard. Analyses were performed in a batch comprising of samples bracketed with IAEA certified primary reference material, IAEA-CH-6, and in-house laboratory working reference materials, Oasis_Glucose (δ^13^C_VPDB_ =  − 10.99 ± 0.03‰) and Oasis_Rice 1 (δ^13^C_VPDB_ =  − 27.67 ± 0.04‰). For the nitrogen isotope analysis, 3.5 mg of the powdered samples were loaded in tin capsules and analysed using the same set up described above. The results are expressed as delta (δ) values in per mil (‰) relative to N_2_ in air. The standards used for nitrogen isotope analysis included the IAEA certified primary reference material, IAEA-NO-3, and in-house laboratory working reference material, Oasis_Rice 1 (δ^15^N_Air_ = 4.1‰). The analytical reproducibility obtained based on repeat analysis of standards were 0.05‰ for the δ^13^C_org_ and 0.1‰ for the δ^15^N_org_ signatures. δ^18^O values for the water samples collected from all three saltern ponds were analysed using the procedure described in Rahul et al.^62^.

## Results

### Microbial mat community

In general, the microbial mat samples showed high richness and diversity. Over 2200 distinct operational taxonomic units (OTUs) from over 90 phyla were identified in all of the samples. The average read counts for all samples was 47,882. Sequencing data are available through NCBI SRA portal with accession number PRJNA595607. Interactive Krona plots that were developed for the samples are accessible through the link, https://github.com/vrnpaul/Saltern-Krona-data, whereas screenshots of relevant Krona plots are provided in the supplementary information.

On average, up to 0.9% of the reads, which passed the quality and filtering steps did not have any assignment based on the SILVA database mapped against. We found that both the white and green bottom (BOT) samples were uneven, with the latter showing the least evenness, dominated by few taxa. On the other hand, the top and BTZ samples, irrespective of white or green was similar throughout. With respect to the abundance demonstrated by Chao1 index, only the white mats had reduced diversity, specifically in the DG and BOT sections (Table S1). The values of these indices were calculated relative to the other layers within the system samples. On average, when the richness and evenness are similar, the value for the index is around 4. As this value increases, richness and evenness also increase correspondingly.

When going deeper into the taxonomic classification, majority of the mat samples had a large number of uncultured, unclassified or candidate phyla in both archaeal and bacterial members. For example, in the White-TOP sample, approximately 34% of the entire archaeal representation (9% of the whole sample) was within the archaeal class WSA2. Similarly, among the *Chloroflexi* phylum in the Green-TOP section, more than 90% of its members (~ 15% of the entire sample) were either uncultured or candidate taxa belonging to classes or lower taxonomic categories. This phenomenon was observed in both mat samples at all stratigraphic levels, suggesting the abundance of potentially novel microorganisms and associated metabolisms within the mats.

**Figure 3:**
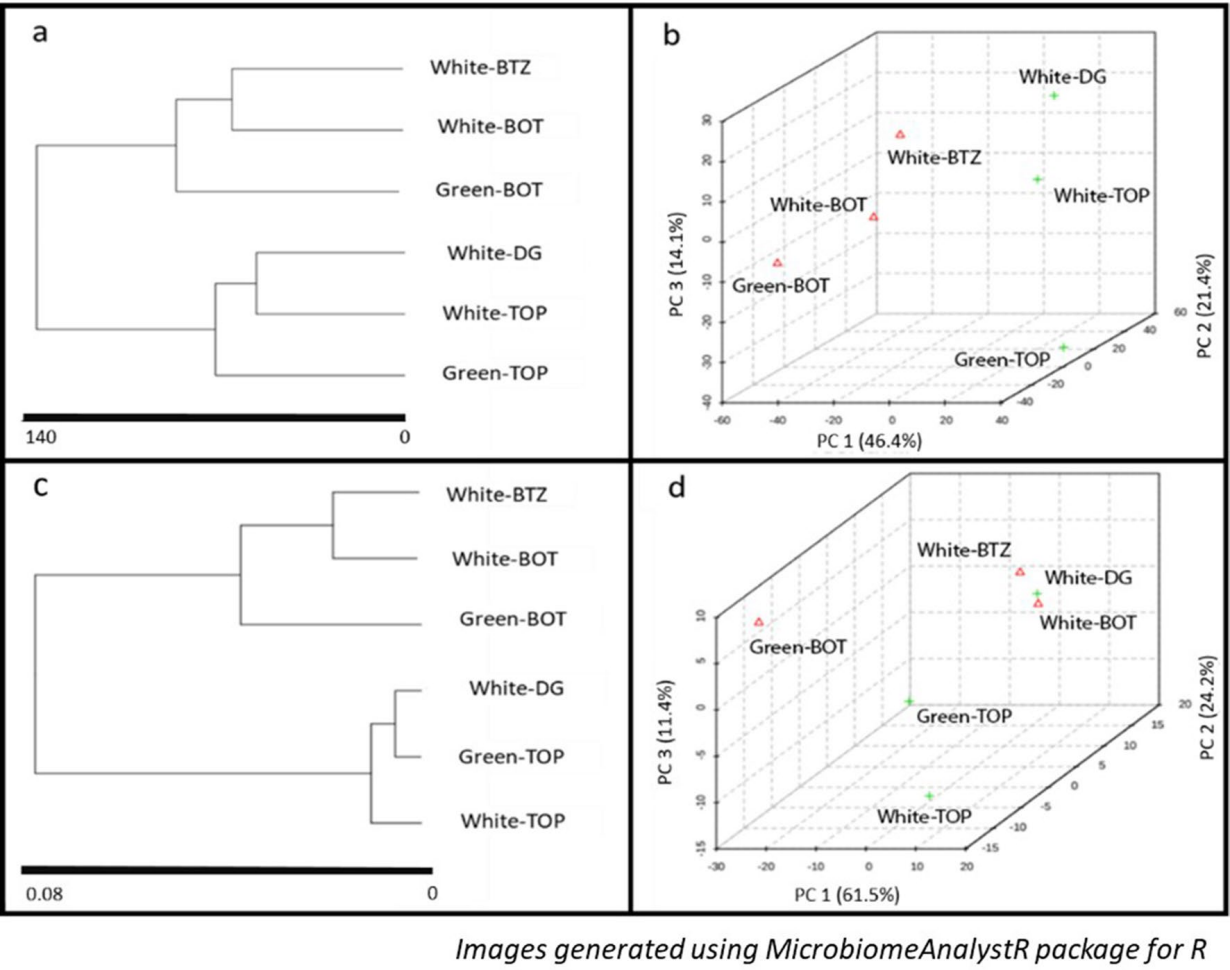
Dendrogram showing the hierarchical clustering of the microbial population (**a**) and predicted metabolic pathways (**c**). The scale shown in (**a**) and (**c**) represent the branch lengths, estimated as a function of the similarity between the samples based on Euclidean distances, and clustering the samples with Ward’s clustering algorithm. The 3D plots based on the Bray–Curtis dissimilarity matrix depict the variability across the selected principal components for the microbial population (**b**) and predicted metabolic pathways (**d**). The explained variances are shown in brackets in (**b**) and (**d**). Images generated through MicrobiomeAnalystR package (https://github.com/xia-lab/MicrobiomeAnalystR) built for R^121^.

The clustering of the samples based on the microbial population was slightly different from the predicted KEGG metabolic pathways (Fig. 3a,c). While the White-BOT and White-BTZ were grouped together, the Green-BOT was more independent, and had an intermediary clustering to Green-TOP. As expected, White-TOP and White-DG, which had similar microbial compositions were clustered together, while the next closest link for these two samples was Green-TOP. Principal component analyses of both the samples echoed the same grouping pattern as revealed by the dendrograms (Fig. 3b,d). The clustering is further highlighted in the heatmap (Fig. S1), showing some of the microbial groups that exhibited high similarity within the mats. The composition of the microbial mats is discussed below with respect to their location, i.e., top, middle (dark green) or bottom, in both types of mats.

#### Top layer

**Figure 4:**
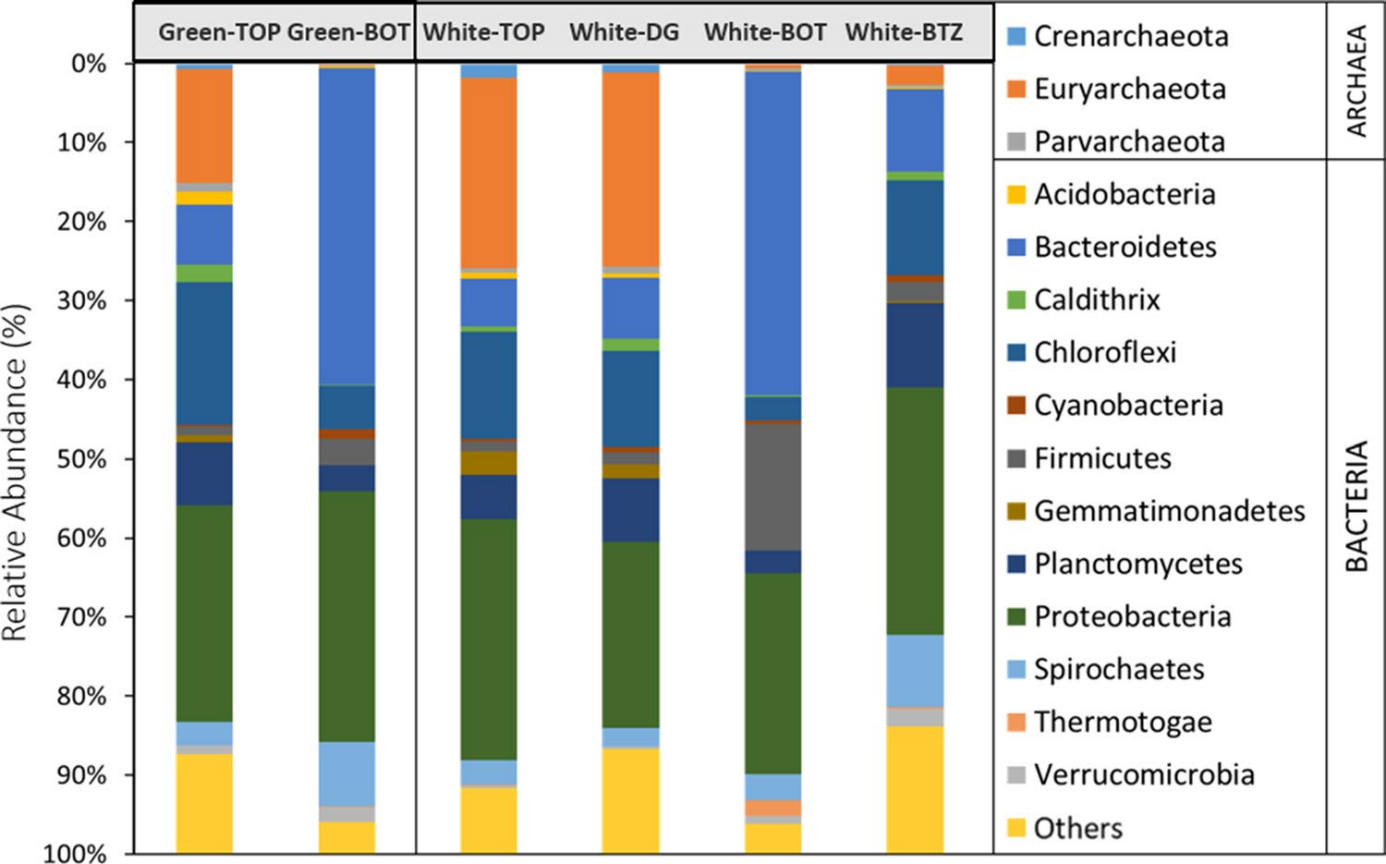
The distribution of phyla in the microbial mat samples showing the various archaeal and bacterial members based on their relative abundance. The green and white mats are shown in adjacent boxes. TOP: top portion of the mat; DG: Dark Green (middle); BOT: Bottom; and BTZ: additional bottom section extracted from the white mat.

The top layers (Green-TOP and White-TOP) had a higher representation of archaeal members when compared to the lower regions. The percentage representation of archaea in the top layers of white and green mats were 27% and 17%, respectively (Fig. 4). On average, WSA2 (34%) and *Euryarchaeota* (50%) were the dominant members within the archaea in the mats. Halophilic *Halobacteriaceae* that are members of *Euryarchaeota* occupied ~ 10% of the entire microbial population (Figs. S2 and S3). The order, *Thermoplasmatales*, which include anaerobic, acidophilic, marine benthic organisms, made up 12% of all archaea. Many of the halophilic members under the family *Halobacteriaceae* were uncultured (only had isolates with sequencing information in the NCBI database), but included *Salinigranum*, and *Halanaeroarchaeum*. The anaerobic, sulfate-reducing bacteria, *Halanaeroarchaeum sulfurireducens* is a one of the few cultured members of the latter^63^. *Crenarchaeota* was the other archaeal phylum that was present more than 1% in the top layers.

All of the Proteobacterial members, except for Campilobacterota, were similar in abundance and dominant (> 33%) in both the top mats (Figs. S2 and S3). Several sulfur-metabolizing bacterial members, such as *Thiohalospira*, *Thiohalorhabdus*, and *Halothiobacillaceae*, which are part of the *Gammaproteobacteria* (11% and 13%, respectively, in green and white mats), were present, whereas majority of sulfate-reducers (60–80%) including *Desulfobacterales*, *Desulfarculaceae*, *and Desulfovibrionales* constituted the entire class *Deltaproteobacteria* in this section of the mats. The ratio of the percentages of the classes, *Chloroflexi*, *Planctomycetes* and *Bacteriodetes*, were similar in both mats, 2.25:1:1.

#### Middle layer (dark green)

The dark green (DG) layer in the green mat showed low reads and repeated sequencing attempts did not provide any better results. Therefore, results from Green-DG are not elaborated here, but are included in the supplementary information (Table S2). In terms of the general microbial population, the middle DG section of the white mat was identical to the top layer; the archaeal population was dominated by WSA2 (10%) and *Euryarchaeota* (14%) and was similar in relative abundance (27% overall) to the top section (Figs. 4 and S4). The other minor archaeal phylum included *Woesearchaeota* (2%). Among the *Proteobacteria* members, *Delta*- and *Gamma*- subgroups were dominant (~ 10% each), with about 4% *Alphaproteobacteria*. Sulfate-reducing bacteria belonging to the orders, *Desulfovibrionales*, *Desulfarculaceae*, and *Desulfobacterales* were represented the most (totaling 7% of the total mat) within the Deltaproteobacterial subgroup. The other major *Deltaproteobacteria* was *Bradymonadales* (2%), which generally includes many microorganisms found in marine sediments. Among the Gammaproteobacterial representatives, *Chromatiales* were dominant (3% overall) and comprised of the family *Halothiobacillaceae* (2%), which includes halotolerant, mesophilic, and obligate chemolithoautotrophic organisms^64^. Many halophilic, sulfur-oxidizing bacteria, such as *Thiohalobacter* and *Thiohalorhabdus* were also present. *Xanthomonadales* was minor (1%), but includes one of the largest groups of bacterial phytopathogens^65^. *Alphaproteobacteria* included *Rhodospirillales* (2%) and *Rhodobacteraceae* (1%), the former representing many purple non-sulfur bacteria. *Anaerolineaceae* was a major family (11%) within the phylum, *Chloroflexi*, some members of which have been shown to be closely associated with methanogenic bacteria and in other hypersaline microbial mat systems^66^. Known hypersaline microbial mat community under the class, *Phycisphaerae* (6%), were also identified. These microorganisms have been found to be associated with marine algae^67^. The final major phylum in the dark green section is *Bacteroidetes* (8%), within which, the common environmental order *Sphingobacteriales*, was dominant (3%). Other significant bacteria that were present in the DG sample include *Firmicutes* (2%), which contains several halophilic species common in hypersaline mats; iron-respiring bacteria, *Deferribacteres*; soil bacteria, *Gemmatimonadetes*; and many other anaerobic phyla, such as *Parcubacteria*, *Spirochaetales* and *Aminicenantes*.

#### Bottom layer

In the bottom sections of the white (White-BOT) and green mats (Green-BOT), the archaeal members were very low (≤ 1.0%), whereas in the bottom-most White-BTZ mat, it was slightly higher (3.0%) (Figs. 4, S5–S7). This percentage of archaea (0.4–1.0%) was much lower when compared to the top and dark green layers (up to 27%). The overall phyla in White-BOT and Green-BOT were similar, but the lower taxonomic ranks were compositionally different.

The phylum, *Bacteriodetes* was dominant in the Green-BOT sample (40%), followed by *Proteobacteria* (32%), *Spirochaetales* (8%), and *Chloroflexi* (5%). *Firmicutes* and *Planctomycetes* were present about 2% each. Interestingly, the photosynthetic *Cyanobacteria* (1%) was only detected in the Green-BOT and White-BTZ sections of the mats. In the White-BOT samples, *Firmicutes* was more abundant (16%) than in the Green-BOT, while other phyla were less than 3.0%. The chemoheterotrophic organisms associated with marine alga, *Phaeodactylibacter*, were as much as one-fourth of all the microbial population in the Green-BOT, but were not detected in the White-BOT. A marine species, *Limibacterarmeniacum*, was detected in the White-BOT and represented about 8% of the overall community. Other major *Bacteriodetes* members detected in the White-BOT included the anaerobic family, *Marinilabiaceae* (8%); the *phylotype* E6aC02, detected in hypersaline endoevaporitic microbial mats; and several other minor representatives of marine microbial members. *Deltaproteobacteria* in both white and green bottom mats were similar in their abundance (~ 5%), but *Alphaproteobacteria* was 5.25 times more in the green mat than white, and included the orders *Rhodobacteraceae*, *Caulobacterales* and *Rhodospirillales*. Conversely, *Gammaproteobacteria* was twice as much in the white than green mats. Thiosulfate-reducing bacteria of the genus *Fusibacter* was significant (10%) in the White-BOT sample, while the hypersaline genus, *Halanaerobiales* occupied around 3%. This sample also had *Campylobacterales* (5%), *Oceanospirillales* (2%), *Chromatiales*, *Marinobacter* (2%), and several sulfate-reducing bacterial groups, such as *Desulfovibrionales* (2%) and *Desulfobacterales* (1%) belonging to *Deltaproteobacteria* (5%). Other important phyla in the White-BOT mat include 2–3% each of *Spirochaetales*, *Thermotogae*, *Planctomycetes*, and *Chloroflexi*.

The White-BTZ section composition was different from the White-BOT; for example, the phylum *Bacteriodetes* was much less (10%) in the White-BTZ, while *Chloroflexi* and *Planctomycetes* were higher (~ 10% each) than the White-BOT (2% each) (Fig. 4). Interestingly, a phylum uniquely associated with the well-studied, hypersaline Guerrero Negro mats, GN01, was detected (9%) in the bottom-Z sample^43^. *Proteobacteria* was the dominant phylum in this Sect. (31%). *Oceanicaulis*, a member of *Alphaproteobacteria* isolated from marine algae^68^, was a genus unique to BTZ sample (3%) and was not detected in other samples. *Verrucomicrobia*, which includes several soil methane oxidizers was identified in all of the bottom samples with a 1–2% representation^69^. The remaining microbial taxa were similar to the bottom and top mats, though varying in their relative percentages.

### Metabolic capacity analyses

**Figure 5:**
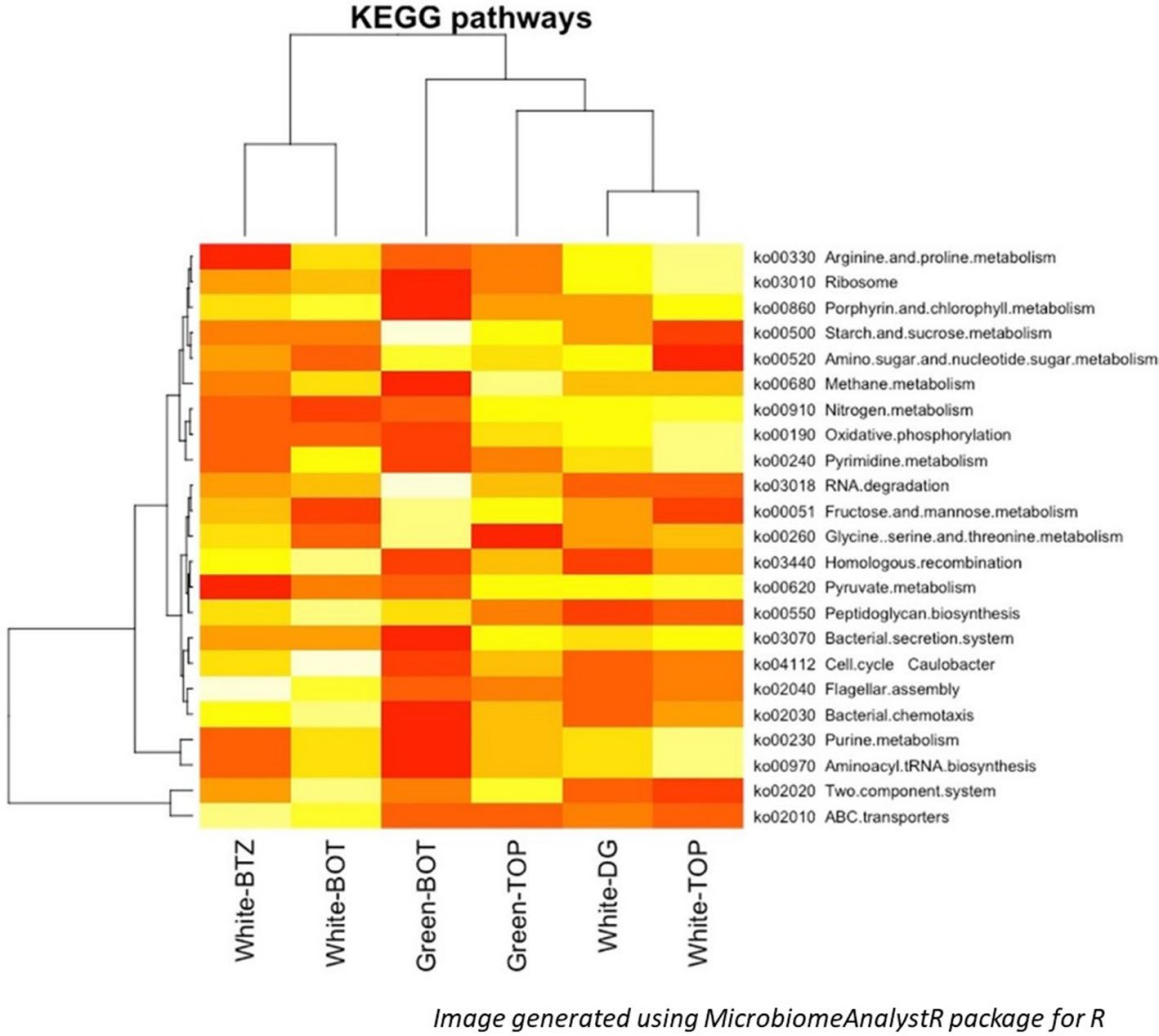
Sample clustering and the top 25 most detected predicted metabolic pathways are depicted in the Heatmap based on Euclidean distance measures, and Ward’s clustering algorithm. Each row represents a single KEGG orthology, while the samples are arranged in columns. The ‘ko’ numbers in the ID are codes for individual orthologs generated in the program, while the second half of the description refers to the metabolic categories at the level-3 annotation within the KEGG database. Images generated through MicrobiomeAnalystR package (https://github.com/xia-lab/MicrobiomeAnalystR) built for R^121^.

Tax4Fun package was used to evaluate the potential metabolic pathways associated with the OTU data. The values discussed below represent the relative abundance of the potential metabolic pathways, identified by mapping to the level 3 KEGG orthology database, within each sample. This is determined by extrapolating the taxa information to the nearest-known neighbor in the bacterial reference database, from which the metabolic potential is estimated, based on the availability of a complete genome. The relative abundance values discussed are normalized using the square-root transformation for easier comparison, and ranged from zero to a maximum of 8.8. Due to the insufficient sample size, we would like to note that significance of the heatmaps showing the predicted metabolisms cannot be tested in our present study. The most dominant pathways predicted are the ATP-binding cassette transporters (ABC transporters), and the two-component regulatory system (Fig. 5). These two mechanisms were dominant in all samples but were slightly higher in the top and middle (DG) samples (7.6 ± 0.6 and 8.2 ± 0.5, respectively). Other major pathways postulated for all samples include conventional microbial mechanisms such as bacterial chemotaxis, flagellar assembly, nucleotide repair, RNA degradation, carbon fixation pathways, and several amino acids and bioproduct (e.g., arginine and proline) syntheses. Oxidative phosphorylation, the final step involved in the production of energy in aerobic organisms was more prevalent in the top sections of the mat than the bottom.

Methane and nitrogen metabolism were two important functions that were predicted based on the microbial population and were possibly associated with members of archaea, such as archaeal class, WSA2 and bacterial phyla *Firmicutes* and *Bacteroidetes*. Both methane and nitrogen metabolisms were slightly higher (2.0, and 2.1, respectively) in the top samples of both mats than in the bottom samples (1.6 and 1.9, respectively). All samples were enriched in pyruvate metabolism (< 1.0), with a slightly highly proportion in the top and middle samples than the bottom, signifying its importance as a key nutrient. The importance of pyruvate, but its lesser known role in hypersaline environments has been suggested earlier^70^. While porphyrin and chlorophyll metabolism indicate active photosynthetic metabolisms, the process of photosynthesis itself was not enriched (< 0.3). Interestingly, sulfur metabolism was very low in the KEGG pathway analysis results. There was no noticeable change in the metabolic pathways moving from the top and deeper into the sample.

### Isotopic signatures

**Figure 6:**
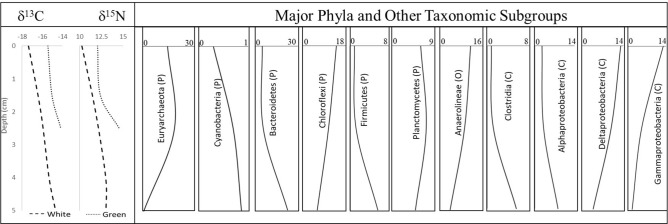
Depth-wise changes of δ^13^C and δ^15^N (‰) isotopic signature along with the major phyla (P) and other taxonomic subgroups (C, Class; O, order). The isotopic values for both white and green mats were chosen from the top, mid-DG, and bottom (Bot) sections at depths 0–1.5 cm, 1.5–2.5 cm, 2.5–5 cm, respectively. Similarly, the community representation was taken as an average of both white and green mats, with the values indicating the percentage within each taxa and not the percentage of the total sequences.

Stable carbon and nitrogen isotopic ratios were measured in the white and green mat, sampled at the same vertical sections as before. The carbon and nitrogen isotopic values in the organic matter for the different sections of the mats revealed distinct depth-wise trends (Fig. 6). For the white mat, δ^13^C_org_ and δ^15^N_org_ values increased from − 17.38 to − 14.68‰ and 10.27 to 13.06‰, respectively. For the green mat, δ^13^C_org_ values varied from − 15.46 to − 14.18‰, and δ^15^N_org_ values from 12.06 to 14.57 ‰. A progressive increase of the stable carbon and nitrogen isotope values with depth was observed. For the white mat, the rate of increase (per cm depth) was 0.90‰ and 0.93‰ for δ^13^C_org_ and δ^15^N_org_ signatures, respectively, while for the green mat, it was 0.64‰ and 1.25‰ for the δ^13^C_org_ and δ^15^N_org_ signatures, respectively. A cross plot between δ^13^C_org_ and δ^15^N_org_ values shows a strong positive correlation (R^2^ = 0.89) (Fig. S8). Comparing the salinity values with the δ^18^O signatures of the water collected from different ponds showed a positive correlation (Fig. S9).

## Discussion

### Depth-wise changes in microbial population

Several uncultured, candidate phyla were identified in both mats indicating that novel strains and even genus or family-level organisms were present. The white and green mats had some unique microbial communities when explored depth-wise. Both mats were taken from the reservoir ponds of the saltern under moderate saline conditions (53 PSU). The White-Top, White-DG and Green-TOP clustered close together both in terms of microbial population and predicted metabolic pathways, suggesting that the top layers and the dark green layer in the middle may be a thick continuous unit, compositionally (Figs. 2, 3, S2–S4). However, the top layer was visually and texturally different from the middle, dark green layer. The white mat, being slightly thicker and located at a higher gradient of the reservoir pond, was exposed above the water surface during peak summertime before the samples were collected, and likely got dry. The white coloration on the top could also be reflective of temporary nutrient depletion associated with bleaching^71^. The middle layer of the green mat (green-DG) did not produce sufficient microbial coverage of the sample even with repeat analysis; however, the top and bottom sections of both white and green mats grouped together and had similar abundance (Figs. 3 and 4). It is therefore highly likely that the middle sections also were similar in composition in both mats. It is unknown as to why this particular sample did not yield sufficient microbial coverage, but the possibility of PCR-inhibitors potentially affecting the library preparation cannot be discounted.

In contrast to many salterns, the Tuticorin facility does not use ocean water (~ 35 PSU) directly for its salt production; rather they rely on the more concentrated subsurface brine water (~ 50 PSU), because of its higher salt content. However, the proximity of the ocean to these salterns and its influence can be observed in the microbial mats, as several marine members were identified in the population analysis. For example, *Bradymonadales*, a *Deltaproteobacteria*, and microorganisms commonly found in marine sediments of the class, *Phycisphaerae*, were abundant in the top and bottom layers of both mats (Figs. S2–S7). Though the mats were isolated from the moderately saline reservoir ponds, many hypersaline microbial groups were also detected, suggesting that a spatio-temporal salinity ‘microgradient’ exists within the mats. In both mats, a decrease in the average values of the salinity was reported (Fig. S10), confirming the presence of a salinity gradient within the mat. During extreme summer days, salinity can rapidly rise leading to salt precipitation especially on the top layers of the mat and triggering more growth and dominance from the hypersaline members. Rainfall or introduction of new brine water can subsequently lead to relative dilution and cause the other members to become more active. Two lines of evidence from the microbial population and predicted functions can be called upon to support this trend. Firstly, the archaeal halophiles, *Halobacteriaceae*, and thermophilic *Thermoplasmatales* are more abundant on the top of both green and white mats (Figs. S2 and S3). A hypersaline bacterial member from a previously studied microbial mat of the class, *Phycisphaerae*, was also found to be present in the top layers and negligible in the bottom layers^72^. Secondly, the concentration of halophilic subgroups in the top layers could be the reason why arginine and proline metabolisms were more pronounced here (Fig. 5). Accumulation of proline has been shown to provide tolerance for microorganisms subjected to high salinity^73^. While overall changes in microbial populations and adaptations due to fluctuating salinity on a large spatial extent are reported in previous literature^22,74,75^, we report a previously unknown phenomenon where a salinity gradient can exist even within the microbial mat.

### Methane, nitrogen and other predicted metabolisms

Certain predicted metabolic pathways coincided with the population structure observed in our samples; though as stated earlier, care should be taken while inferencing Tax4fun data exclusively for functional interpretation due to its potential limitation outside of human datasets^60^. For example, the archaeal class, WSA2, and the phylum Euryarchaeota, include methyl-reducing methanogens^76^, and were significant in the top surface of the mats (Figs. S2–S4). Correspondingly, methane and nitrogen metabolisms based on the predicted pathway analyses were slightly higher in the top sections of the mat than in the middle or bottom (Fig. 5). In addition to archaea, bacterial groups, such as the order *Anaerolineaceae*^77^, were dominant in the top sections of the mat (~ 11%), while it reduced to 2% at the bottom sections of the mat (Fig. 6). Some members of *Anaerolineaceae* has been shown to be a dominant member providing essential organic acids to methanogenic archaea^66,78^. *Alpha*-, *Delta*- (including several sulfate-reducing bacterial members), *Epsilon*- and *Gamma*- *Proteobacteria* had significant representations in all layers of the mat. Nitrogen fixation could have likely been performed by members of *Gamma and Delta- Proteobacteria*, which are highest in the top and middle layers^79,80^. *Gammaproteobacteria* have earlier been shown to possess a high amount of ammonia-oxidation (amoA) genes in similar hypersaline mats^81^. Nitrogen fixation is also performed by a variety of bacterial phyla, including *Firmicutes*, *Bacteroidetes* and the archaeal phylum *Euryarchaeota*, all of which are present richly in the top layers (Fig. 4,^82^). *Crenarchaeota*, another minor archaeal phylum found in the top layers, has been shown to have members that play an important role in nitrogen cycle, especially ammonia oxidation^83^.

The prediction of the ATP-binding cassette transporters (ABC transporters), and the two-component system was expected as it is common to most microbial systems (Fig. 5). The ABC transporter group is involved in energy generation and transport of related compounds and molecules in the cell and surrounding environment. The two-component system, consisting of a sensor protein-histidine kinase (HK) and a response regulator (RR), enables bacteria to sense, respond, and adapt to changes in their environment or in their intracellular state. Flagellar assembly pathway detected in all samples suggest that the microorganisms could be involved in active movement within the mats^22^. The suggested presence of oxidative phosphorylation, which is used by aerobic organisms, likely indicates that oxygen-based metabolisms are prevalent in the top and middle sections of the mat. Contrastingly, the anaerobic, sulfate-reducers in the *Deltaproteobacteria* were 11% on average over all layers, while the top mats alone had an average of 5% of this class. This trend could possibly imply that anaerobic members could also exist in the upper layers of the mat. This phenomenon was reported in other hypersaline mats as well, where sulfate-reducers not only survived in the oxic layers, but also had the capacity to respire oxygen^84–86^. Conversely, some aerobic members (for example, *Limibacter armeniacum*^87^ was prominent in the bottom mat and not in the top (Fig. S6). The complex distribution of the aerobic and anaerobic populations within the different layers suggests that oxygen can have important effects on influencing the local microbial populations.

Surprisingly, sulfur metabolism was not strongly predicted based on the 16S rRNA gene sequencing, with only minor (0.5) relative abundance of sulfur metabolism and sulfur relay systems^88^, though several sulfate-reducers such as *Desulfovibrionales*, *Desulfarculaceae*, and *Desulfobacterales* and sulfur oxidizing bacteria, such as *Thiohalobacter* and *Thiohalorhabdus* were detected in all layers. As explained earlier, sulfate-reducers within the *Deltaproteobacteria* ranged from 1–10% of the overall mat composition, signifying that it is an important group in the microbial mat. The lack of sulfur metabolism predicted through the analysis is a reminder that the interpretation from some bioinformatics tools should be treated with care, and not all functional pathways can be positively identified. This is likely because the Tax4fun program is suitable for human samples, and could suffer in its predictive abilities when it is applied for environmental samples^60^. Additionally, the presence of the microorganisms alone does not mean that they were active, and functional capabilities can change rapidly in a dynamic mat system^89–91^. Another drawback of the predictive metabolism method is that we are currently identifying the pathways as being at level 3 KEGG orthology. This could include the possibility that the analysis did not capture essential genes, such as a key methanogenic gene (K00399, methyl-coenzyme M reductase), which could be detected by level 4 orthology, where the resolution of individual genes involved in the pathways is increased, thereby aiding inferences. However, due to the limitations of the software used, we are unable to verify the level 4 orthology at this point. Whole-genome shotgun metagenomics and metatranscriptomics required to address this depth of analysis were not in the current scope of this study, and will be addressed and validated in future studies.

### *Cyanobacteria* and eukaryotic algae: comparison with mats from other regions

Several oxygenic and anoxygenic phototrophs were reported to be present in varying abundances in the mats. For example, *Cyanobacteria* though present, was extremely limited, with the highest abundance (1.2%) in the Green-BOT mat. *Cyanobacteria* tend to migrate downwards into the mat to protect themselves from harmful UV radiation^46^. Since *Cyanobacteria* was limited, it is possible that eukaryotic algae were one of the primary producers in the system. Though, we did not specifically test for eukaryotes in the mat, the abundant identification of several bacterial members (*Phycisphaerae*, *Phaeodactylibacter* and *Oceanicaulis*) that have been shown to be associated with marine alga could indirectly indicate the presence of the eukaryotic phototrophs^67,68,92^. However, such an inference should be treated with caution since no direct observation of eukaryotes were made. Eukaryotic algae have been detected in other hypersaline microbial mat systems^93^. Contrasting evidences on the effects of salinity on *Cyanobacteria* have been reported ranging from no effect^94^ to having a negative effect on the population^95^. Whether salinity has a role in the dominance of algae *versus Cyanobacteria* in microbial mats is unknown. Microalgae and eukaryotic algae richness have been reported in several salterns throughout the world, even in Tuticorin^37,96^. It is possible that cyanobacterial populations would be more dominant in the ponds with higher salt concentrations (condenser and crystallizer), as they are better adapted to hypersaline conditions than eukaryotes^45^. However, at salt concentrations more than 250 PSU, they become limited, and the primary production is typically taken over by the unicellular eukaryotic green algae, *Dunaliella*^96^. This influence of salinity on the bacterial and eukaryotic phototrophs needs to be explored more in the context of other ponds in the salterns.

Compared to one of the most well-characterized hypersaline mats found at Guerrero Negro (GN), the microbial mats at the Tuticorin salterns did not have a distinguishing horizontal profile^24,43^. However, some of the general patterns in the phylogenetic distribution was observed. The archaeal distribution was one of the key similarities noted^23^, where archaea was concentrated in the upper layers of the mat. Contrastingly, *Cyanobacteria* was very limited (≤ 1.2%) in the Tuticorin mats, whereas these were much more abundant (10–35%) in the GN mats (24). Furthermore, even the small cyanobacterial population was found only in the bottom layer, opposite to the trend seen in GN^43^. *Firmicutes*, *Chloroflexi*, *Bacteriodetes*, *Planctomycetes* and *Proteobacteria* were prominent phyla identified in mats from both environments (Fig. 4). Particularly, *Gamma*- and *Delta*- *Proteobacteria* in mats from both locations showed a decrease with mat depth.

Similar microbial profiling studies in Israel and Slovenia were performed from mats collected from the condenser and crystallizer ponds. In the mats obtained from the Sečovlje salterns of Slovenia, the bottom sediments called ‘petolas’ were isolated and their microbial community characterized^44^. *Cyanobacteria* was found to be dominant in the top few layers. However, Gammaproteobacterial population was also dominant and decreased with depth. Cyanobacterial dominance in the top layers of the mats were found in the gypsum crusts of a commercial saltern in Eilat, Israel^71^. Plenty of other phototrophs, including anaerobic members were reported in that study. One common linkage in all of these saline to hypersaline settings is the abundant presence of sulfate-reducers, signifying its ubiquitous role in these ecosystems^85,94^.

### Relating carbon and nitrogen isotopic ratios to population and metabolism

Carbon and nitrogen concentrations, and their isotopic values in organic matter reveal important information about the biogeochemical processes that operates during microbial mat formation and development^14,25,31^. While both isotopic ratios can be considered in terms of individual microbial groups or specific mechanisms, it is important to note that these signatures are the result of net metabolic activities occurring in the different layers^28,97^. In GN microbial mats, for example, variability in the δ^13^C_org_ signatures were indicative of alternating dominance by phototrophs, whereas the bulk signature was shown to be overprinted by the heterotrophic activities of sulfate-reducers and methanogens^97^. In addition, physicochemical conditions and any post-sedimentation modifications are discounted in interpreting the values^98,99^.

In a hypersaline setting, methanogenic bacteria typically produce organic matter with lighter isotopic compositions (both C and N). This process governs the observed covariance of both carbon and nitrogen concentrations and their isotopes. Microbial mat from microbialites growing in similar hypersaline environments showed near identical trends^100,101^. In our study, a strong correlation between δ^13^C_org_ and δ^15^N_org_ values was documented, similar to the peritidal stromatolites of South Africa (Fig. S8,^101^). Any scatter in the relationship shows that the carbon in the mats has been transported from the continental region, rather than in-situ production. However, a strong correlation between carbon and nitrogen isotopes, as shown in this study, indicates the absence of transported organic C and N material within the mat systems. Conversely, it can be inferred that the carbon and nitrogen in the salterns are authigenic, i.e., sourced locally, and that these components are actively cycled within the various microbial groups.

The δ^13^C_org_ value also reflects the type of carbon in the organic matter present in the mat. The depth-wise increase in the carbon isotopic composition is mainly driven by a change in productivity. In marine or hypersaline sediments, the deeper sections are more alkaline in nature due to bicarbonate ion production that would have driven the enrichment of δ^13^C_org_ values with respect to the top layers. The ^15^ N enrichment of organic nitrogen values at the bottom layers as compared to the top layers indicates enhancement of denitrification process with depth. Analysis of the δ^15^N_org_ signature in similar microbial mats also showed a small increase in the isotopic values with depth^25^.

The amount and type of microbial taxa play a pivotal role in controlling the carbon and nitrogen cycling within the mats and are in turn reflected in the stable isotope ratios. The changes in carbon and nitrogen isotopic compositions can be compared with the change observed in microbial populations with depth (Fig. 6). Interestingly, *Euryarchaeaota* and WSA2 showed a decreasing trend with depth indicating that a decrease in the methanogen population (and in turn methane production) resulted in a relative enrichment of the carbon isotope signal. *Bacteriodetes*, *Firmicutes*, *Clostridia* and *Alphaproteobacteria* subgroups followed a similar trend to the carbon and nitrogen isotope signatures. On the other hand, *Chloroflexi*, *Deltaproteobacteria* and *Gammaprotebacteria* showed reverse trends. Similar to many methanogens, *Deltaproteobacteri*a, dominated by sulfate reducers, decreased with depth.

Salinity further plays an important role in determining biogeochemical processes. For example, nitrogen fixation by *Cyanobacteria* is hindered by increased salinity, but the process can be compensated by members of *Deltaproteobacteria*^102^. The abundance of *Deltaproteobacteria* in the top layers of the mats and its decrease with depth implies that even within the mats, salinity ‘microgradients’ can exist, and in turn influence the overall metabolic processes. Low amounts of *Cyanobacteria* preclude it from being used as a dominant photosynthetic mechanism. On the other hand, anoxygenic phototrophs were commonly identified. Cyanobacteria are known for their extremely variable ^13^C fractionation during photosynthesis, and is predominantly influenced by CO_2_ concentrations^103,104^. It is not known whether the type of carbon fixation mechanism by eukaryotic alga would be different from that of *Cyanobacteria*. Previous work has shown that green algae has a more lighter ^13^C signal than diatoms, and that CO_2_ fixation mechanisms influence the ^13^C patterns^10^. A more thorough knowledge of the dominant phototrophic community will help better characterize the isotopic signatures resulting from its carbon fixing mechanisms. Furthermore, nitrogen fixation through eukaryotic algae can be expected to yield a slightly identical isotopic signature^105^. Non-phototrophic communities, such as sulfate-reducers belonging to *Deltaproteobacteria*^106^, can further deplete δ^15^N signature and could help explain the lower values in the surficial sections. Organic material produced by phototrophs likely play an important role in supporting such diazotrophs (organisms that can fix N_2_), which have δ^15^N values between − 2‰ and 0‰^25,107,108^.

Similar to other salterns throughout the world, the ponds in the Tuticorin salt production facilities have a wide range of salinity, from 50–150 PSU. The δ^18^O signatures of the water collected from different ponds showed a strong correlation with salinity (Fig. S9), a trend seen in other hypersaline settings^109^. Though microbial mats were not found in the ponds with higher salinities, the isotopic patterns reveal that its similarity to other hypersaline systems could help in comparing environmental conditions, and answer questions as to why such mat features are present in some salterns but not in others, and if there is a seasonal pattern to the occurrence of the mats. Such information would be essential in further understanding microbial adaptations, functioning and diversity under hypersaline conditions, and the results could be useful in the interpretation of to paleoenvironmental, paleoclimatic and astrobiological environments. For example, in the search for life on Mars, many briny environments have been identified^110^. Understanding the microbial metabolisms and the signatures they produce (or leave behind) on similar environments on Earth will make us better equipped to explore for life on the red planet. The isotopic signatures and microbial populations identified in modern microbial mats and microbialites can similarly provide clues into past salinity and temperature conditions during Early Earth^111,112^. Fossil evidences suggest that microbial life on Early Earth was tolerant to high salinity and pH conditions, either localized in proximity to hydrothermal vents or evaporative basins, and poorly ventilated with nearly no oxygen, but dozed with excess UV photons^113^. Here, we drew analogy of Early Earth coastal sabkhas hosting microbial mats and biofilms from equatorial region, but with oxygen rich modern atmosphere and comparatively less intense UV radiation. The abundance of biofilm or EPS with well-developed microbial mats was common in such hypersaline systems around mid-latitude, but rarely in the equatorial region with seasonal climate. Such preservation of microbial mats associated with evaporites was common from Archean age as exposed near Pilbara area of Western Australia, which include a mixed carbonate–evaporite–siliciclastic deposit^114^. Thus, microbial and isotopic patterns of their modern counterparts, such as those presented in this study, could provide possible clues to understand the microbial populations that could have existed in the past.

### Limitations of the study

The use of Tax4fun program in our studies, though has been applied to other investigations to varying degree of success, has potential biases, and therefore the results and interpretations of metabolic predictions presented here must be treated with caution. Such programs have been shown to be used more efficiently to predict functions in human samples, but could provide incorrect results when applied in an environmental context^60^. While the functional changes in the different layers of the mat based on the population were predicted to a certain extent, a thorough investigation into more relevant methods such as metagenomics and metatranscriptomics of the mat are needed^115,116^. For example, whole-genome sequencing has been not only shown to be better at identifying phyla and genera^117^, but also at estimating functional genes^118^. Metabolic pathways, such as photosynthesis, nitrogen fixation, sulfate-reduction, and methane production can provide critical data pertaining to the functioning of microbial mats in this study based on detected microbial population^119^. While the metabolic potential was predicted, it can be improved by using more advanced omics analyses.

## Conclusion

The current study presents a case for a cross-disciplinary approach to investigate microbial metabolism in mat systems, and a novel comprehensive report of its kind from the Indian subcontinent. Both bacterial and archaeal members, with several candidate phyla and unknown family level taxa were identified in both white and green mats. Previously uncultured and unclassified members were commonly found, indicating that potentially novel species are yet to be identified from these environments. Spatial distribution among mats showed some unique populations with high richness. The results correlate with similar studies on microbial mats in other salterns and hypersaline settings. On the other hand, *Cyanobacteria* population, which was dominant in similar salterns, was very limited in the Tuticorin reservoir pond mats, suggesting that the role of primary producers could be undertaken by eukaryotic algae, and other bacterial phototrophs. Salinity plays a more important role in controlling microbial richness, diversity and functioning^115^, with possible microgradients existing within the mats. Our results proffer a previously unseen observation that a salinity ‘microgradient’ exists even within a mat, influencing the microbial distribution and possibly functioning. Micro-electrode studies focusing on depth-wise salinity changes in other hypersaline mats need to be conducted to expand and verify this concept. Methane and nitrogen metabolism were major pathways predicted, though the microbial population analysis revealed a more diverse functional pool of microorganisms including oxygenic and anoxygenic phototrophs, sulfate-reducers, and fermenters, to name some.

Recent advances in multi-omics methods including extraction of multiple biomolecules such as DNA, RNA, metabolites, and protein enable the identification of taxa and their respective functions within a system^120^. In future studies, we will employ these methods to identify taxa, their adaptions, and functions within the different layers of the mats. Additionally, we will focus on conducting mesocosm studies including labelled nutrients and characterizing the isotopic values of various components within the mat to understand the relationship between the signatures and the impact of microbial metabolism on nutrient absorption rates across the various depths^25,100^. We will focus on expanding our study to the sediments from the condenser and crystallizer ponds, which exhibit much higher salinity. The influence of salinity both in the micro- and macro-level on the biogeochemical cycling must be functionally and isotopically characterized to understand the changes and adaptations of the microorganisms over a larger spatiotemporal extent.

## Supplementary information

Supplementary Information.

## Data Availability

All data were collected through the research activities conducted by the authors. Sequencing data are available through NCBI SRA portal (Accession Number PRJNA595607). All other data are provided in the text or supplementary information in the form of tables or graphs. Individual krona plots of the microbial data along with full OTU aligned and taxonomic assignment information are shared in github (https://github.com/vrnpaul/Saltern-Krona-data).
