# Supplementary material for: Depthwise microbiome and isotopic profiling of a moderately saline microbial mat in a solar saltern
Source: Sci Rep. 2020 Nov 26;10:20686. doi: 10.1038/s41598-020-77622-w (PMC7693307; doi:10.1038/s41598-020-77622-w)
Supplement: Supplementary file 1 — Supplementary Information. [file 41598_2020_77622_MOESM1_ESM.docx]

***Supplementary nformation***

***Depthwise Microbiome and Isotopic Profiling of a Moderately Saline Microbial Mat in a Solar Saltern***

Varun Paul^1*^_,_ Yogaraj Banerjee^2,3^, Prosenjit Ghosh^2,3^, Busi Susheel Bhanu^4^

^1^Department of Geosciences, Mississippi State University, Mississippi State, MS 39762, USA

^2^Centre for Earth Sciences, Indian Institute of Science, Bangalore, India

^3^Interdisciplinary Centre for Water Research, Indian Institute of Science, Bangalore, India

^4^Luxembourg Centre for Systems Biomedicine, University of Luxembourg, Esch-sur-Alzette, L-4362, Luxembourg

*Corresponding Author, email: [vgp25@msstate.edu](mailto:vgp25@msstate.edu)

Table S1. Observed richness, Chao and Shannon diversity indices for the samples.

| **Sample** | **Observed**  **(Richness)** | **Chao1** | **Shannon** |
| --- | --- | --- | --- |
| Green-TOP | 2086 | 2223 | 6.1 |
| Green-BOT | 1929 | 2036 | 5.1 |
| White-TOP | 2476 | 2570 | 6.0 |
| White-DG | 1270 | 1439 | 5.9 |
| White-BOT | 1164 | 1288 | 4.7 |
| White-BTZ | 2037 | 2176 | 6.0 |

Table S2. Major microbial groups in the mid-section of green mat sample, Green-DG (Dark Green)

| **Phyla** | **Class** | **Order** | **Family** | **%** |
| --- | --- | --- | --- | --- |
| Chloroflexi | Anaerolineae | Anaerolineales | Anaerolineaceae | 14.3 |
| Firmicutes | Clostridia | Clostridiales | Lachnospiraceae | 42.9 |
| Tenericutes | Mollicutes | Mollicutes RF9 | Uncultured bacterium | 42.9 |


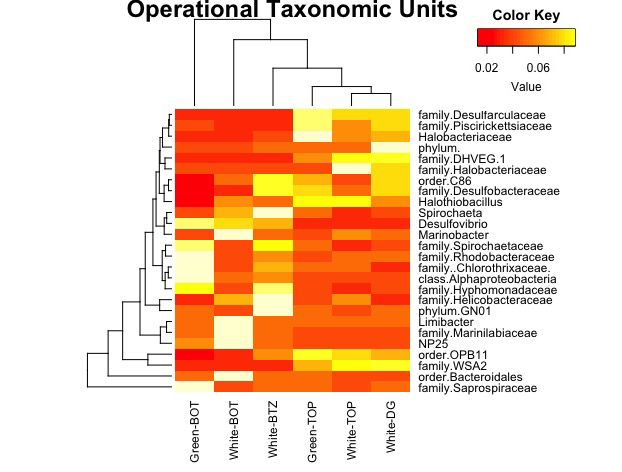
**Fig. S1** Heatmap based on Euclidean distance measures, and Ward’s clustering algorithm depicts the hierarchical clustering across samples and the top 25 most detected microbial taxa. The rows highlight individual taxonomic annotations, with the samples arranged in columns.


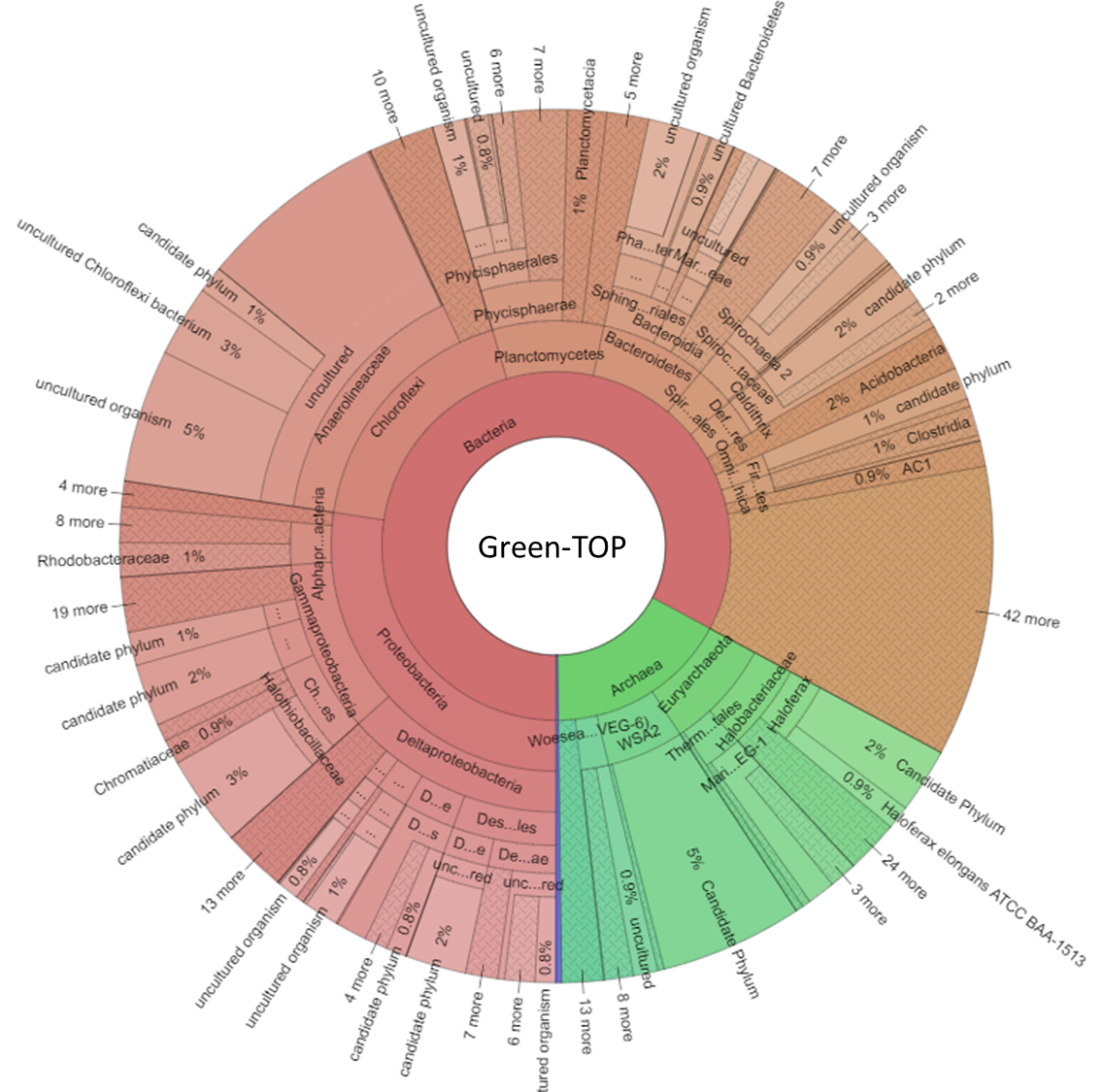


**Fig. S2** Krona plot showing the microbial diversity of the TOP section of the green mat. The relative abundance of individual taxa within each sample are shown.


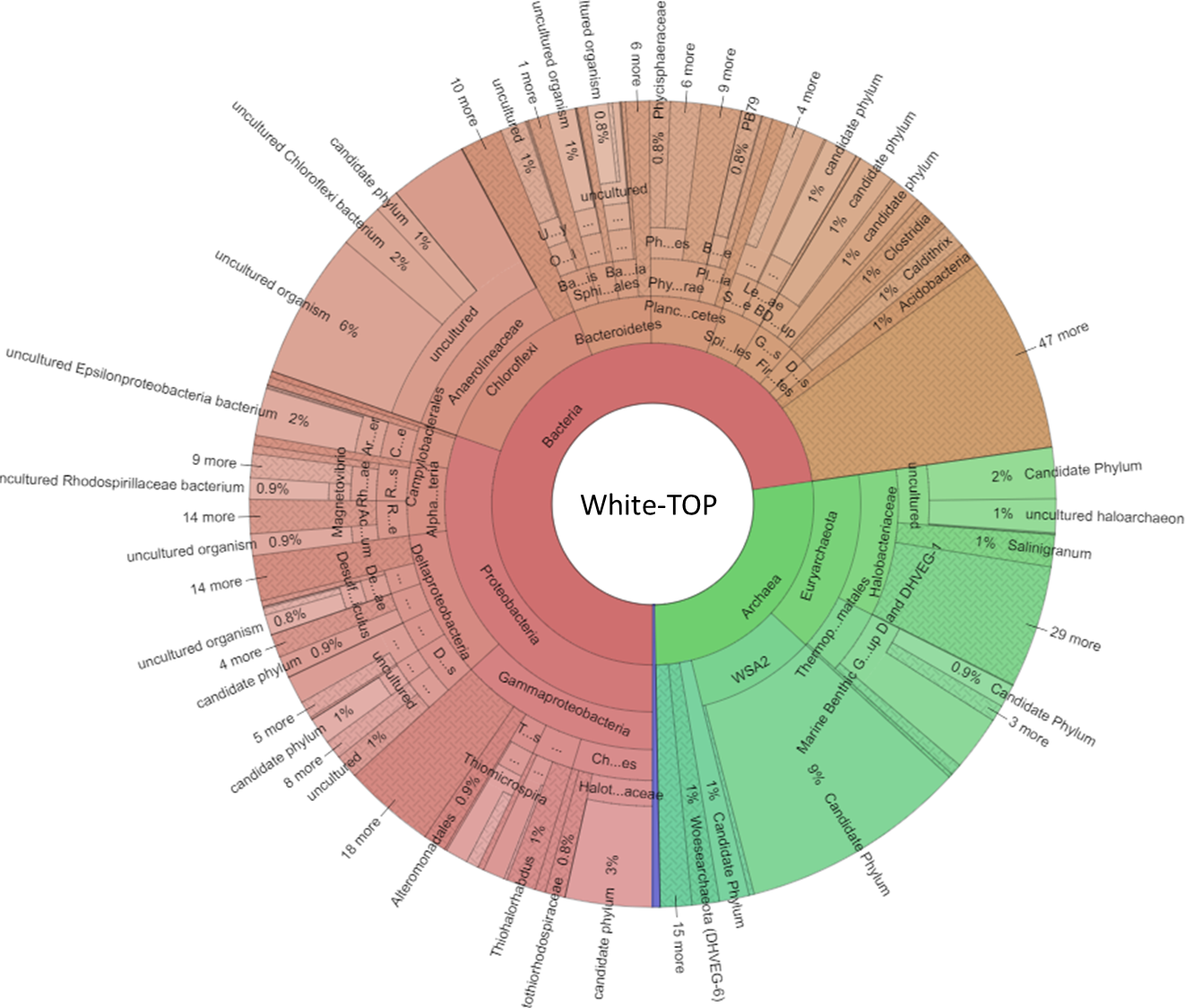


**Fig. S3** Krona plot showing the microbial diversity and their respective relative abundances within the White-TOP section


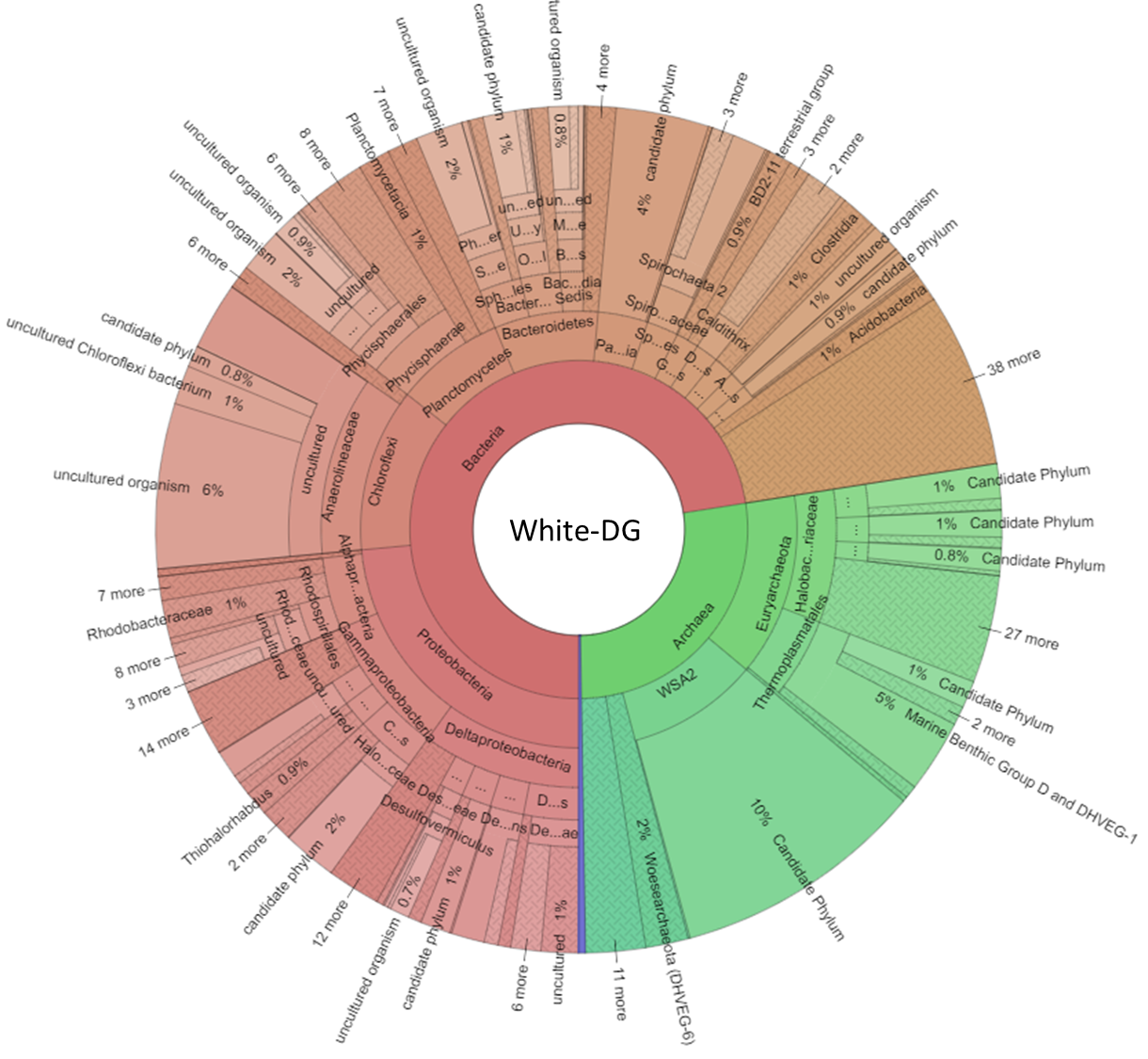


**Fig. S4** Krona plot showing the microbial diversity and relative abundance of the White-DG (middle) section


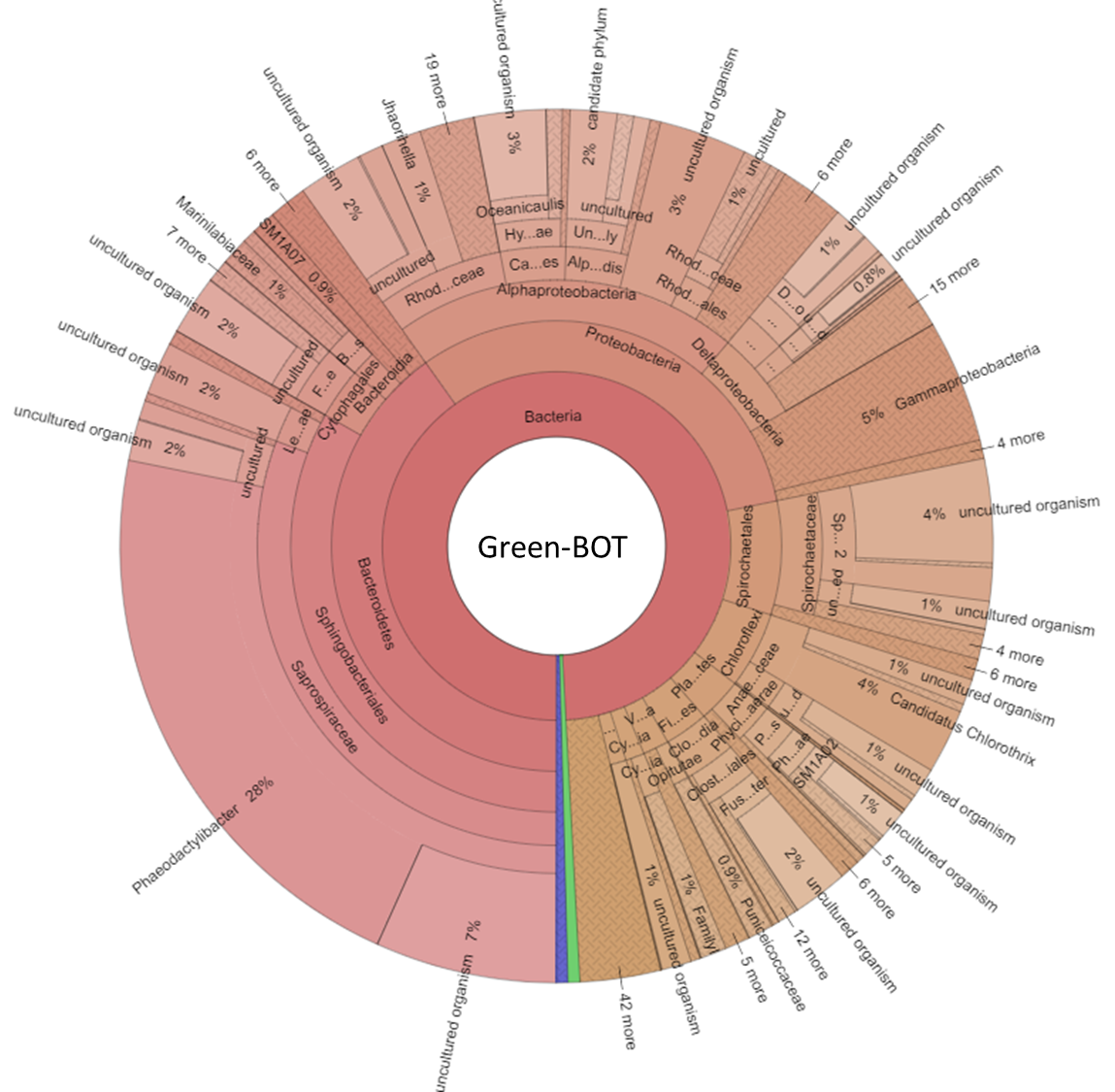


**Fig. S5** Krona plot showing the relative abundance and microbial diversity of the BOT (bottom) section of the green mat


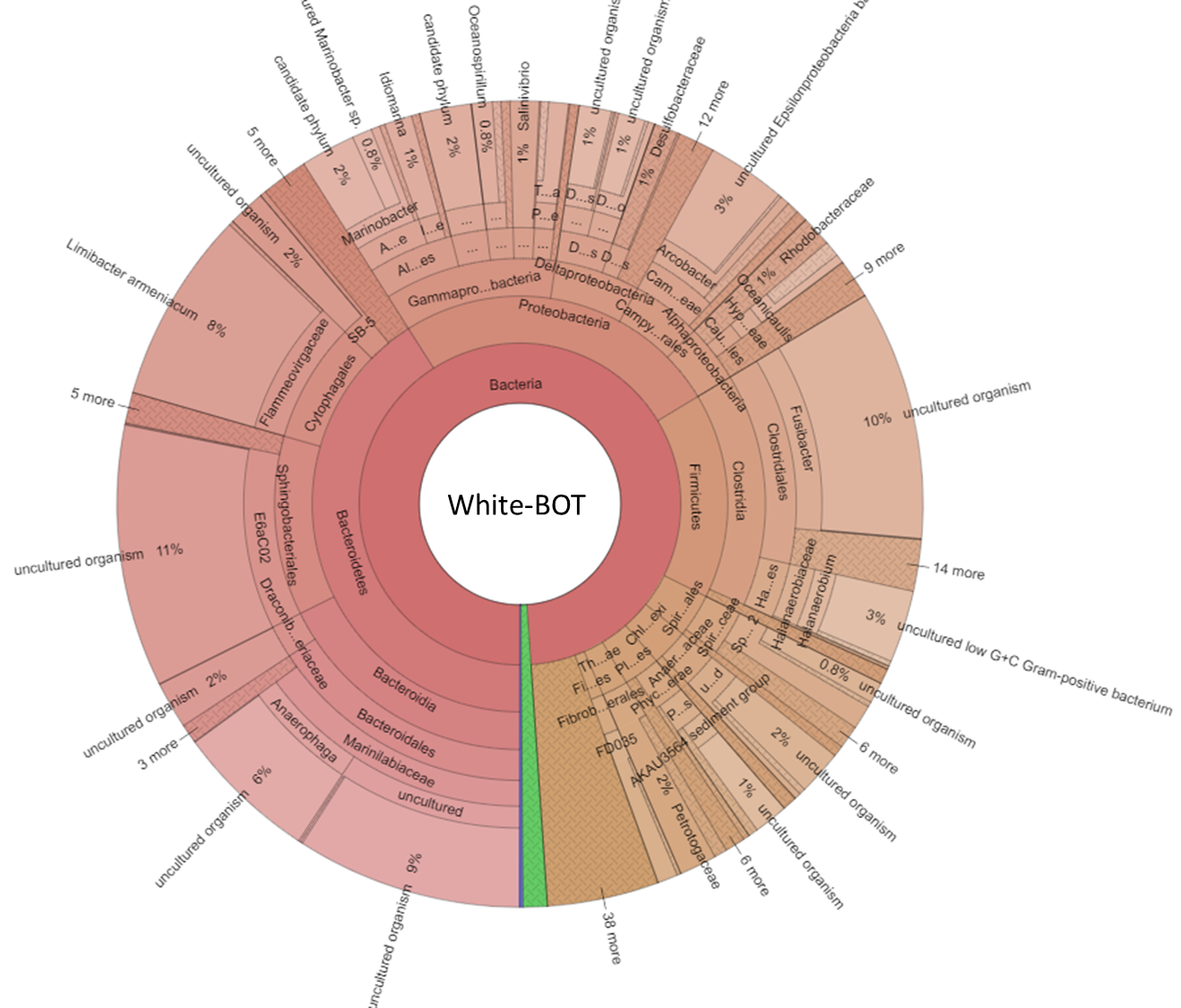


**Fig. S6** Krona plot showing the microbial diversity of the White-BOT (bottom) section


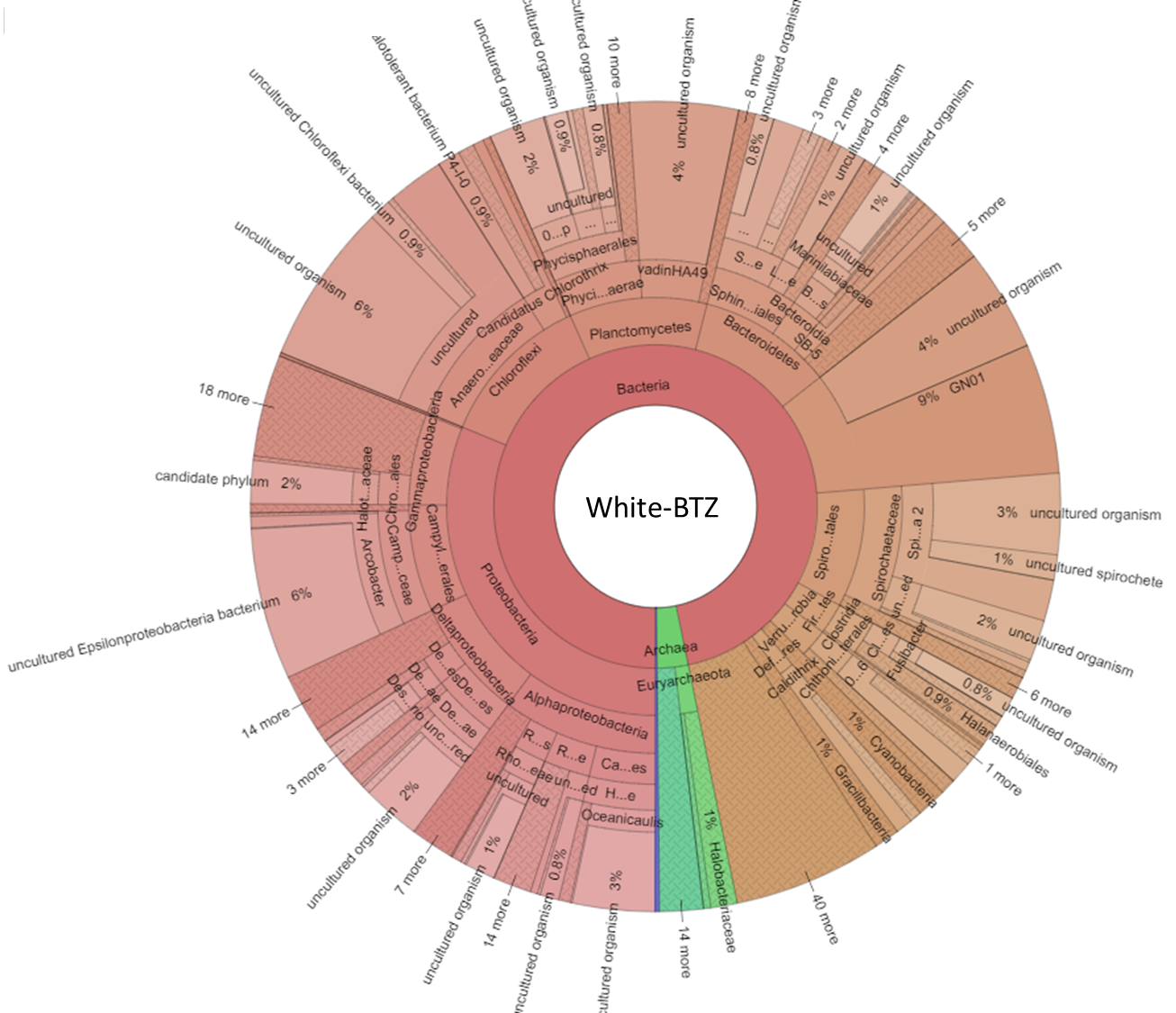


**Fig. S7** Krona plot showing the microbial diversity of the White-BTZ (additional bottom) section


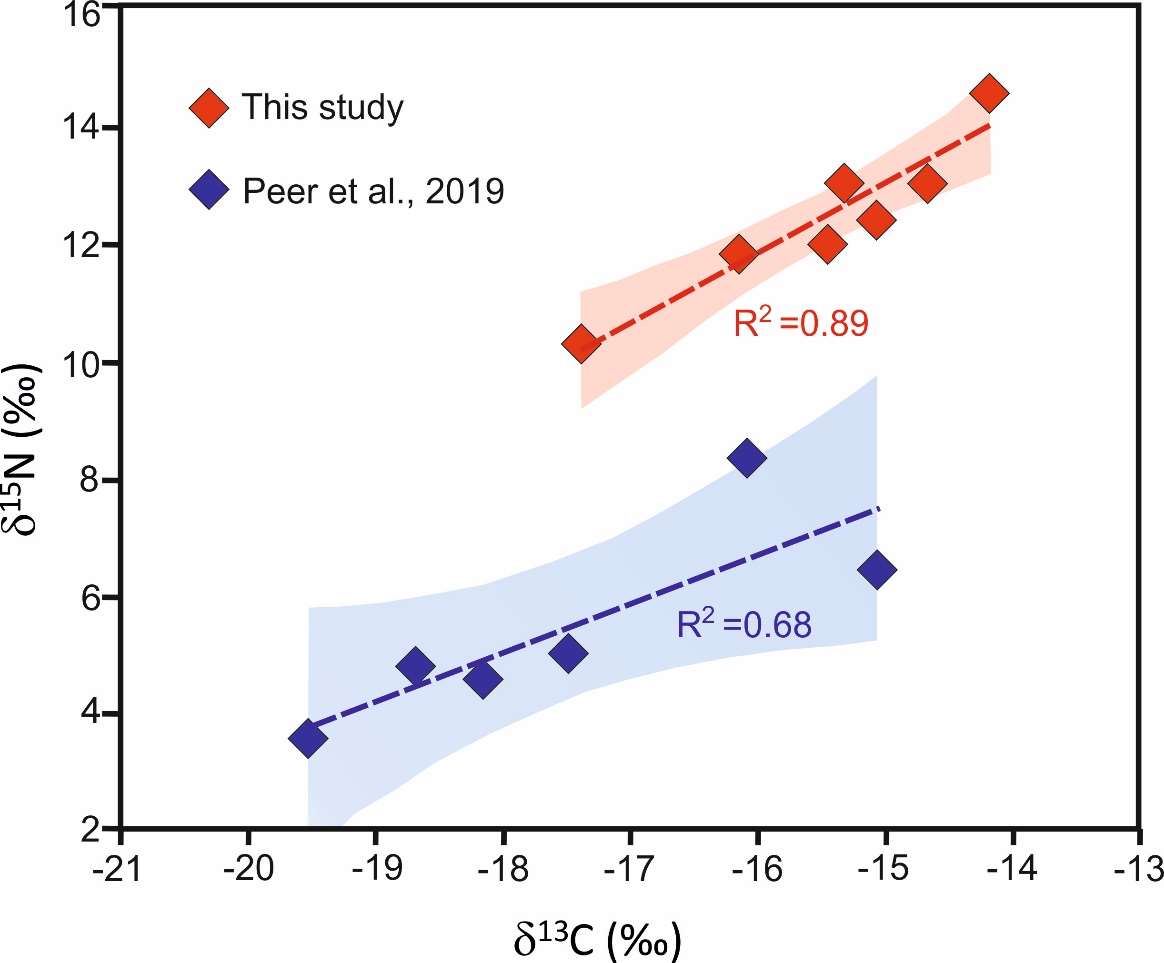


**Fig. S8** A δ^13^C_org_ vs δ^15^N_org_ cross plot shows strong correlation for the mats investigated in the present study (orange dashed line). A similar correlation was observed (blue dashed line) for a modern peritidal stromatolite from South Africa reported by Peer et al. (2019)


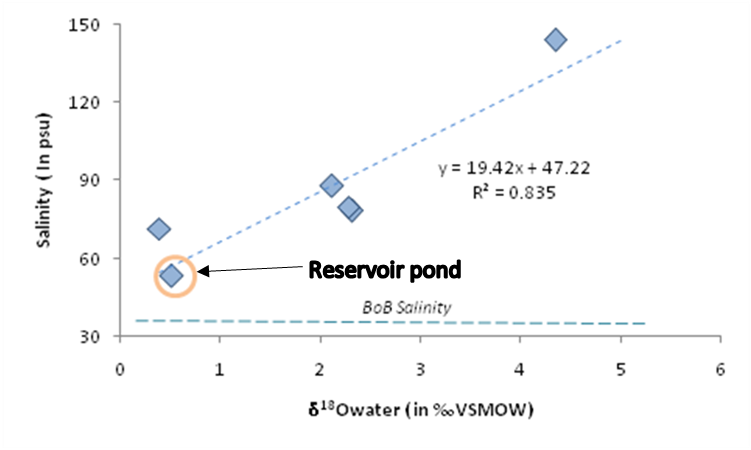


**Fig. S9** Salinity (PSU) and δ^18^O_water_ (in ‰ VSMOW-Vienna Standard Mean Ocean Water) relationship for the various ponds at the Tuticorin saltern determined in the present study. The circled data point indicates the reservoir pond from which the mat samples were collected. Other data points are from the condenser and crystallizer ponds which correspondingly have higher salinities. BoB=Bay of Bengal, used to represent ocean salinity.

**
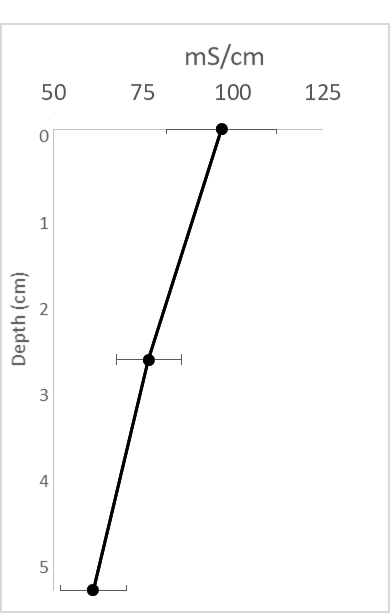
**

**Fig. S10** Average conductivity values (mS/cm) measured in the white and green mats. Error bars for the top, mid and bottom samples of both mats are provided.
